# TRIM8: Making the Right Decision between the Oncogene and Tumour Suppressor Role

**DOI:** 10.3390/genes8120354

**Published:** 2017-11-28

**Authors:** Mariano Francesco Caratozzolo, Flaviana Marzano, Francesca Mastropasqua, Elisabetta Sbisà, Apollonia Tullo

**Affiliations:** 1 Institute of Biomembranes, Bioenergetics and Molecular Biotechnologies, IBIOM-CNR, Via G. Amendola, 165/A-70126 Bari, Italy; mf.caratozzolo@ibiom.cnr.it (M.F.C.); f.marzano@ibiom.cnr.it (Fl.M.); francesca.mastropasqua@gmail.com (Fr.M.); 2 Institute for Biomedical Technologies ITB, CNR-Bari, Via G. Amendola, 122/D-70126 Bari, Italy; elisabetta.sbisa@ba.itb.cnr.it

**Keywords:** TRIM8, tumour suppressor, oncogene, stemness, innate immunity, p53, NF-κB

## Abstract

The TRIM8/GERP protein is a member of the TRIM family defined by the presence of a common domain structure composed of a tripartite motif including a RING-finger, one or two B-box domains, and a coiled-coil motif. The *TRIM8* gene maps on chromosome 10 within a region frequently found deleted and rearranged in tumours and transcribes a 3.0-kB mRNA. Its expression is mostly ubiquitously in murine and human tissues, and in epithelial and lymphoid cells, it can be induced by IFNγ. The protein spans 551 aa and is highly conserved during evolution. TRIM8 plays divergent roles in many biological processes, including important functions in inflammation and cancer through regulating various signalling pathways. In regulating cell growth, TRIM8 exerts either a tumour suppressor action, playing a prominent role in regulating p53 tumour suppressor activity, or an oncogene function, through the positive regulation of the NF-κB pathway. The molecular mechanisms underlying this dual role in human cancer will be discussed in depth in this review, and it will highlight the challenge and importance of developing novel therapeutic strategies specifically aimed at blocking the pro-oncogenic arm of the TRIM8 signalling pathway without affecting its tumour suppressive effects.

## 1. TRIM8: A Representative Member of the Large TRIM Family of Proteins

**Figure 1:**
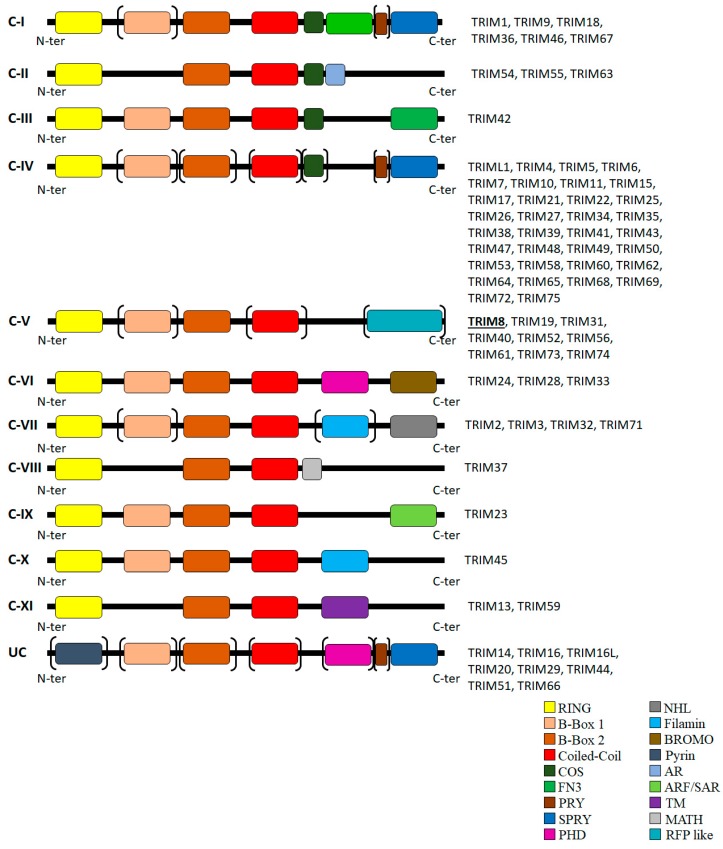
Classification of human TRIM proteins. The TRIM protein family is composed of 11 sub-families, from C-I to C-XI. Some of the TRIM proteins remain unclassified (UC), since they do not have a RING finger domain, unlike the classified TRIM proteins. Additional domains are: NHL: NHL repeats; COS: COS box motif; FN3: fibronectin type III motif; PHD: plant homeodomain; BROMO: bromodomain; MATH: meprin and TRAF homology domain; TM: transmembrane domain; AR: acid-rich region; RFP like domain. The domains in brackets indicate that they can be absent.

TRI-partite Motif 8 (TRIM8) is a member of the TRIM family of proteins, which is composed of a plethora of protein-encoding genes in humans. The TRIM family members, also named RBCC proteins, are characterized by the presence of a tripartite motif whose striking structural feature is the rigid conserved pattern, the order and combination of the domains Really Interesting New Gene (RING) finger (R), one or two zinc-finger motifs named B-box (B) that are highly similar to the RING domain, and an associated Coiled Coil region (CC) (Figure 1) [1,2]. These motifs are an integrated functional structure, rather than a collection of separate modules, and this minimal structure is selectively maintained to carry out a specialized common basic function of the protein family members. All TRIM proteins contain the RING domain and a BBox2 domain, while some also contain a BBox1 domain which differs in its spacing of zinc-binding residues. The C-terminal domains found in TRIM proteins are diverse and, on their basis, the TRIM family members can be classified into 11 sub-classes (Figure 1) [3,4].

With the exception of a few members that share a high degree of aminoacid conservation, the entire family is characterized by a relatively low primary sequence homology. Only the cysteine and histidine residues that define the RING and B-box patterns and the hydrophobic residues in the coiled-coil region are highly conserved and serve to maintain the domain scaffold, whereas the intervening sequences have rapidly evolved to acquire novel specificity and physiological functions. 

Phylogenetic analyses have indicated that the TRIM proteins have been under heavy selection pressure and that the number of genes may have arisen from species-specific duplication. Lower invertebrates ranging from flies to sea squirts (Ascidiae) have a low number of TRIM genes (6–10 genes), whereas the nematode *Caenorhabditis elegans* has 18 genes; jawless fish (lampreys) still have relatively low numbers of TRIM genes, whereas all jawed fishes and mammals have expanded families of >60 TRIM genes and, in humans, over 80 members have been identified. The chicken genome only holds 37 TRIM genes, even though they are evolutionarily closer to mammals than fish [5].

The different domains of TRIM family proteins regulate cellular localization and higher order structures and are involved in several functional activities, mainly by shuffling their binding partners, as well as exhibiting a role in enzymatic regulation via molecular interaction or dominant-negative effects. 

Most of the TRIM family proteins have also been defined as E3 ubiquitin or ubiquitin like molecule ligases (SUMOylation and ISGylation) [6,7,8,9], suggesting their main role in the regulation of cellular protein stability and tuning [10,11] (Figure 1). In particular, the RING domain, a Zinc finger C3HC4 type, initially predicted to be involved in protein-protein interaction [7], endows the E3 ubiquitin-protein ligase activity. Indeed, mutations of C or H residues determine, in most cases, the loss of this functional role. E3 ubiquitin-protein ligase activity is likely to be a general function of this domain and various RING fingers also exhibit binding to E2 ubiquitin-conjugating enzymes (UBCs [12]).

TRIM/RBCC proteins are involved in a broad range of biological processes, and have important roles in differentiation, development, intracellular signaling, protein quality control, autophagy, and immune responses, by regulating various signaling pathways. Furthermore, many TRIM proteins are induced by type I and type II interferons (IFNs), suggesting that TRIM proteins have an important role in anti-viral and anti-microbial systems [13,14,15]. Mutations in the genes encoding certain TRIMs have been associated with human diseases, classified as immunological diseases, or developmental disorders [13]. Moreover, several TRIM members are involved in cancer either as tumour suppressors genes or as oncogenes, by controlling a broad range of processes including transcriptional regulation, cell proliferation, apoptosis, DNA repair, and metastasis [16]. 

Recently, one of the TRIM members, TRIM8, has been emerging as a “double-edged weapon”, since contrasting data is reported on its role either in sustaining uncontrolled cell proliferation or in counteracting the same process as a tumour suppressor gene. Nevertheless, TRIM8 is involved in other cellular functions tightly related to cancer, such as inflammation and innate immunity.

TRIM8 gene maps on chromosome 10q24.3, within a region mostly involved in deletion or rearrangements in glioblastomas. Therefore, it was initially designated as a glioblastoma expressed RING finger protein (GERP) [17]. The TRIM8 gene transcribes a 3.0-kB mRNA, which is expressed in various human tissues, including brain, lung, breast, gut, placenta, kidney, muscle, and germinal center B cells [18].

**Figure 2:**
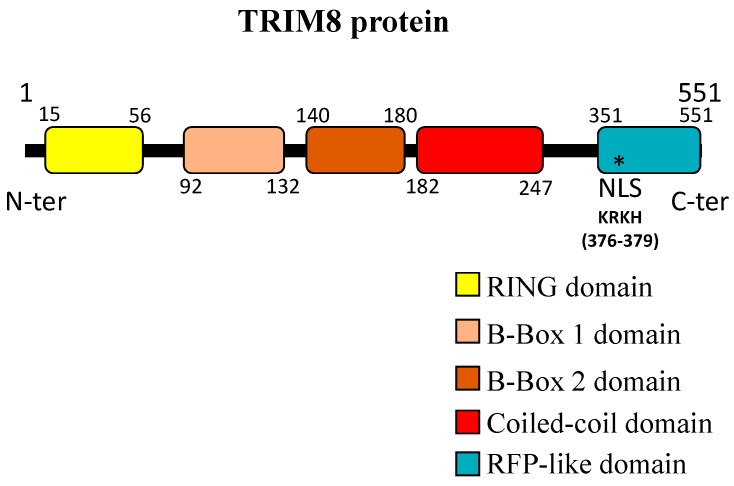
TRIM8 protein structure. The different TRIM8 domains are reported with the numbers indicating the first and the last aminoacid for each one.

The protein is composed of 551 amino acids (aa) with a molecular weight of 61.5 kDa, contains a Nuclear Localization Signal (NLS), and forms specific nuclear structures similar to the TRIM19/PML Nuclear Bodies (NBs) (Figure 2). These nuclear structures depend on the coiled-coil domain, since the deletion of this domain induces diffused nuclear staining and no discrete foci. Interestingly, the deletion of the C-terminus showed distinct cytoplasmic staining. This data suggests that TRIM8 modulates the activity of important cellular proteins through protein-protein interactions mediated mainly by the coiled-coil and the C-terminus domains [19].

## 2. The Role of TRIM8 in Tumorigenesis: Tumour Suppressor or Oncogene?

### 2.1. The Tumour Suppressor Role of TRIM8

Involvement of TRIM8 in cancer was first highlighted in brain tumours including glioblastomas, where the TRIM8 loss of heterozygosity was observed. This was also later observed in other cancers such as clear cell Renal Cell Carcinoma (ccRCC) [17,20]. In a transcriptome-wide analysis of Larynx Squamous Cell Carcinoma (LSCC), the most frequent neoplasm of the head and neck, TRIM8 down-regulation was found to be associated with metastatic progression suggesting its tumour suppressor role. Moreover, TRIM8 downregulation was also found in other tumours as Glioblastoma Multiforme (GBM), clear cell Renal Cell Carcinoma (ccRCC), anaplastic thyroid cancer (ATC), colorectal cancer (CRC), Chronic lymphocytic leukemia (CLL), and osteosarcoma cell lines [19,20,21,22,23,24,25,26]. Accordingly, the restoration of TRIM8 levels in osteosarcoma U2OS cells stably inhibited their capability of forming colonies, confirming the anti-proliferative action of TRIM8 and suggesting its role in cell-cycle control. Recently, some important findings have provided the first mechanistic link between TRIM8 and the *p53* tumour suppressor gene [21]. Indeed, it was demonstrated that TRIM8 is a p53 direct target gene, and by a positive feedback loop, TRIM8 is able to potentiate the p53 tumour suppressor activity, controlling the molecular switch that directs p53 toward the transcriptional activation of cell cycle arrest and DNA repair genes, such as *p21* and *GADD45A*, leading to the suppression of cell proliferation. To further strengthen these recent findings, TRIM8 was shown to exert its anticancer activity through a joint action that provides, on one hand, the activation of the p53 tumour suppressor role and, on the other, the degradation of MDM2, the main negative regulator of p53 stability [21]. By employing TRIM8 deletion mutants, it has been demonstrated that the TRIM8-RING domain alone (42 aa) is necessary and sufficient for inducing p53 stabilization, p53-dependent inhibition of cell proliferation, p53 transcriptional activation, and MDM2 degradation [21]. 

Other members of the TRIM protein family control carcinogenesis by modulating the stability and activity of the p53 tumour-suppressor protein or of its main regulator MDM2 [27]. In particular, TRIM19 (also known as promyelocytic leukaemia protein-PML) is able to support p53-Thr18 phosphorylation in response to DNA damage by recruiting p53 into PML nuclear bodies (NBs), and protecting it from MDM2 degradation, while TRIM13 directly co-localizes with MDM2 in nuclear structures and mediates MDM2 polyubiquitination and degradation with the consequent increase in p53 stability and activity [28,29,30]. Contrary to this, TRIM24, TRIM32, TRIM39, TRIM59, and TRIM66 have been shown to mediate p53-polyubiquitination and degradation, while TRIM29 (also known as Ataxia Telangiectasia group D-Complementing-ATDC) has been shown to bind and antagonize p53-mediated functions [31,32,33,34,35,36]. Altogether, these findings strongly support a close correlation between TRIM family proteins and p53 tumour suppressive functions.

**Figure 3:**
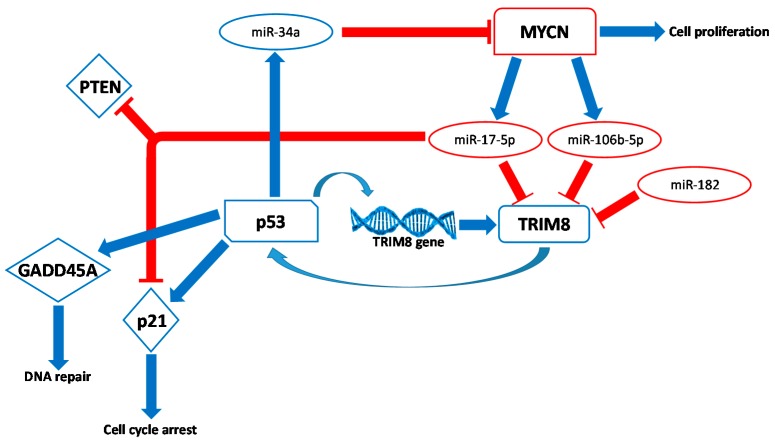
Schematic representation of the tumour suppressor network involving TRIM8. Following cellular stress, the p53 tumour suppressor protein transactivates *TRIM8* gene, which in turn stabilizes p53, promoting the transcription of p53 target genes involved in cell proliferation arrest (*p21*), DNA repair (*GADD45A*), and the suppression of the MYCN oncogenic activity by miR-34a. Without the inhibitory effect of miR-34a, MYCN promotes the transcription of miR-17-5p and miR-106b-5p, which target TRIM8 mRNA, promoting its degradation. Moreover, in anaplastic thyroid cancer, miR-182 targets TRIM8 mRNA, maintaining low levels.

A prominent role for TRIM8 in regulating cancer cell growth was shown in vivo in ccRCC. RCC is a family of cancers including five major subtypes (clear cell, papillary type I and type II, chromophobe, collecting duct, and unclassified RCC) that originate from the renal tubular epithelium, but unlike other epithelial cancers originating from other districts such as the colon, breast, lung, stomach and bladder, p53 mutations in RCC are particularly rare, especially in the ccRCC subtype. ccRCC is the most common subtype of RCC, accounting for about 80% of surgical cases, and is characterized by exceptionally high resistance to radiation and chemotherapy. More than one-third of patients with RCC have evidence of metastases at the time of diagnosis, and approximately 33% develop systemic recurrence after primary tumour resection [37]. Intriguingly, it has been demonstrated that the TRIM8 expression level was significantly decreased in tumour samples compared to non-tumour tissue of the same patients, and such a signature was typical of more malignant renal tumours, whereas it was not observed, for instance, in benign renal oncocytomas (RO) [20]. The TRIM8 deficit, observed in patients affected by ccRCC, was explained by the up-regulation of the miR-17-5p and miR-106b-5p members of the miR-17-92 family, whose overexpression has an oncogenic effect by promoting tumour cell proliferation [38]. Additionally, in human gliomas, a heterogeneous group of primary malignant brain tumours with strong resistance to chemotherapy and radiotherapy, the reduction of TRIM8 expression, correlates with high levels of miRNA-17-5p, leading to an unfavourable clinical outcome of the patients. The miR-17-92 cluster, located in the third intron of the *C13orf25* gene at 13q31.3, contains seven miRNAs, which are over expressed in many cancers. The human genome contains two paralogues of the miR-17-92 cluster: the miR-106b/25 cluster, located on chromosome 7 (7q22.1) in the 13th intron of the Mini-Chromosome Maintenance gene (MCM7), and the miR-106a/363 cluster, located on chromosome X (Xq26.2). While the miR-106a/363 cluster is rarely expressed in adult human tissues, the miR-17-92 and miR-106b/25 clusters are emerging as key actors in a wide range of biological processes including tumorigenesis [39,40,41]. An increasing number of papers report that miR-106b-5p and miR-17-5p, above all the microRNAs of the miR-17-92 family, are overexpressed in many different chemo/radio-resistant cancers, including ccRCC, glioma, CRC, and CLL cell lines [22,38,39,40,41]. It has been demonstrated that miR-17-5p and miR-106b-5p directly target the 3′UTR of TRIM8 and both transcriptionally and post-transcriptionally repress the expression of TRIM8, indicating that TRIM8 and miR-17-5p/miR-106b-5p may be part of the same circuit involved in ccRCC and glioma pathogenesis [20,24]. Interestingly, a high expression level of MYCN transactivates the miRNA 17-92 and miR-106b/25 clusters, which in turn, down modulates other different targets, such as the tumour suppressor p21, PTEN, contributing to tumorigenesis (Figure 3). Interestingly, the inhibition of miR-17-5p and/or miR-106-5p leads to the recovery of TRIM8-mediated p53 tumour suppressor activity, which in turn strongly inhibits MYCN-dependent cell proliferation. Indeed, MYCN is a direct target of miR34a, whose expression is activated by p53. The down-regulation of the MYCN-miR17-92 network represents an interesting approach to inhibit uncontrolled cell proliferation and tumour growth in vivo (Figure 3) [22].

Moreover, in ATC tissues and cell lines, it has been reported that TRIM8 mRNA and protein levels are kept low via the up-regulation of another miRNA, miR-182, which directly targets TRIM8 mRNA (Figure 3) [26].

### 2.2. Targeting TRIM8 as A Therapeutic Approach for Human Malignancies

In addition to conventional cancer intervention strategies, such as surgery, chemotherapy remains one of the most important components of cancer treatment. Although chemotherapy is often capable of inducing cell death in tumours and reducing the tumour bulk, many cancer patients experience recurrence and ultimately death because of treatment failure. Therefore, resistance to chemotherapy is one of the most critical aspects and one of the most difficult challenges for oncological research.

Cancer cells may acquire resistance to chemotherapy, or may have a high basal level of resistance through a variety of mechanisms, which can be summarized as follows: (1) decrease of drug concentration in the cell due to the activation of transporter proteins encoded by Multiple Drug Resistance genes (MDR); (2) increased detoxification of the drug within the cell; (3) increased repair of the damaged target; (4) abrogation of apoptosis or cell cycle arrest, for example, due to the mutation or inactivation of tumour suppressor genes, i.e., *p53*. Since TRIM8 is a p53 regulator, a disruption of its stabilizing function may be included among the mechanisms that may render the p53 protein dysfunctional or inactive, despite the lack of genetic lesions within the *TP53* gene. Indeed, in several tumours, the p53 pathway may be inactivated by alterations in its regulators or by yet unknown mechanisms, leading to resistance to cytotoxic therapies. All these cancers, together with those carrying mutations in the *p53* gene, are associated with chemo-resistance and, in general, predict a considerably worse patient prognosis in comparison with malignancies with functional p53.

Recent results in ccRCC, an aggressive drug resistant cancer showing wild type *p53*, are shedding light on the strong correlation that exists between TRIM8 deficit, p53 inactivation, and chemoresistance. The recovery of TRIM8 expression in ccRCC-derived cell lines was able to induce a great p53-dependent reduction in the proliferation rate, which became more pronounced when the cells were treated with the chemotherapy drugs Nutlin-3 and Cisplatin, but more interestingly, when the cells were newly sensitive to treatment with Axitinib and Sorafenib, two specific drugs currently used in the treatment of many cancers, including ccRCC [20,21].

Coherently with this finding, it has been found that in ATC tissues and cell lines, TRIM8 downregulation is significantly correlated with the upregulation of miR-182, which targets TRIM8 mRNA. MiR-182 overexpression induces cellular growth by repressing TRIM8 expression, greatly contributing to the chemoresistance of ATC cells [26].

Altogether, these findings suggest that chemoresistant tumours can be successfully sensitized to conventional chemotherapy if combined with modalities designed to reactive p53, and more broadly, TRIM8 could be considered a new target for therapeutic intervention in cancers where *p53* is wild type and its pathway is defective.

### 2.3. The Flipside of the Coin: TRIM8 as An Oncogene

**Figure 4:**
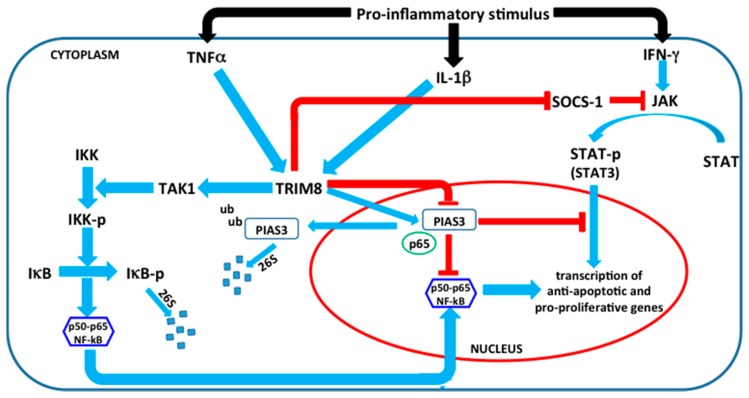
Schematic representation of the oncogenic network involving TRIM8. Proinflammatory cytokines, such as TNFα and IL-1β, promote NF-κB activation mediated by TRIM8, through two different mechanisms: (1) promoting the translocation of PIAS3 from the nucleus to cytoplasm and its subsequent degradation; PIAS3 in the nucleus interacts with p65, preventing NF-κB activation; (2) mediating TAK1 polyubiquitination and subsequent activation. TAK1 is a member of the MAPK kinase kinase family that promotes the phosphorylation of IKK (IκB Kinase) leading to the phosphorylation of IκBα, its degradation, and activation of NF-κB. Moreover, TRIM8 induces the activation of the JAK-STAT pathway promoted by Interferon-γ (IFN-γ), through the degradation of two STAT proteins inhibitors, PIAS3 and SOCS-1 (suppressor of cytokine signalling-1).

Although some studies point to the tumour suppressor role of TRIM8, a number of oncogenic mechanisms have been proposed concerning a TRIM8 role in the regulation of inflammatory pathways, including the NF-κB pathway, and therefore in supporting tumour onset and progression [42,43]. NF-κB represents an interesting link between inflammation and cancer, although the molecular mechanism has not yet been clearly understood [44,45]. Many solid tumours exhibit activated NF-κB that may be the result of either exposure to proinflammatory stimuli in the tumour microenvironment or mutational activation of upstream components in IκB kinase (IKK)–NF-κB signaling pathways. Once activated, NF-κB can inhibit apoptosis, stimulate cell proliferation, and promote a migratory and invasive phenotype that is associated with tumour progression [45,46,47,48,49,50,51,52,53,54,55,56]. NF-κB activation is triggered by the proinflammatory cytokines Tumour Necrosis Factor α (TNFα and by interleukin-1β (IL-1β) and requires the signal-induced phosphorylation of IκB proteins by the IKK complex and its consequent degradation [57,58]. TNF is one of the pro-inflammatory cytokines that is constitutively present in tumour microenvironments and regulates various steps of tumourigenesis. In particular, TNF induced NF-κB activation is a critical regulator of cell survival and death, having implications in many physiological and pathological conditions including cancer. In this cascade mechanism, the role of TGF-β activated kinase 1 (TAK1), a serine/threonine kinase member of the MAPK kinase kinase family, is also important, and phosphorylates and activates IKK transmitting the upstream signal from the receptor complex to the downstream signaling molecules (Figure 4) [59,60,61,62].

In this context, recent studies have proposed TRIM8 as a novel oncogene, because it is involved in the positive regulation of the TNF-induced NF-κB pathway by promoting the translocation of PIAS3 (Protein Inhibitor of Activated STAT-3) from the nucleus to cytoplasm. It has been suggested that TRIM8 may promote PIAS3 ubiquitination via its ligase activity, translocation to cytoplasm, and degradation through the proteasome. PIAS3 interacts with p65 in the nucleus, preventing NF-κB activation (Figure 4) [43,63]. PIAS3 translocation to the cytoplasm could determine the SUMOylation of Rac1, which may stimulate lamellipodia, cell migration, and invasion, as recently observed [64]. The RING domain, responsible for ubiquitin ligase activity, is important for TNF induced NF-κB activation. These findings are further supported by the loss of expression of PIAS3 in many tumours including human gastric carcinoma [65] and glioblastoma multiforme tumours [66].

Another way by which TRIM8 mediates the activation of NF-κB is by TAK1 ubiquitination, enhancing the inflammatory responses (Figure 4) [42]. 

The analysis of cellular localization of TRIM8 strongly suggests that TRIM8 is translocated to the cytoplasm in response to TNF. This translocation is inhibited by nuclear transport inhibitors that negatively regulate NF-κB activation. This suggests that TRIM8 acts at two subcellular sites in the regulation of the NF-κB pathway. In response to TNF, it translocates to the cytoplasm, thus activating TAK1, and may facilitate p65 translocation. TRIM8 again translocates back to the nucleus where it may act on PIAS3. In the nucleus, it may exclude PIAS3 from the nucleus and p65 may become free to activate NF-κB. It is also possible that TRIM8 may act as a carrier protein for PIAS3 translocation and degradation, resulting in NF-κB activation (Figure 4). These interesting hypotheses need further experimental evidence to confirm the multiple role of TRIM8 in the NF-κB pathway.

Finally, another example, in which TRIM8 has been described as an oncogene, concerns its role in decreasing the protein stability of SOCS-1 (suppressor of cytokine signalling-1) and reversing the SOCS-1-mediated inhibition of JAK-STAT activation by Interferon-γ (IFN-γ) [67]. Under physiological conditions in normal cells, the activation of STAT proteins is rapid and transient, because they are negatively regulated by proteins such as SOCS and PIAS [68,69,70]. Since TRIM8 also negatively regulates PIAS3 by degradation, this results in the prolonged activity of STAT3 (Figure 4) [63]. Once activated, STAT3 has a broad range of biological functions, including cell activation, cell proliferation, the protection of tumour cells from apoptosis, and migration by regulating genes encoding antiapoptotic and proliferation-associated proteins, such as Bcl-xL, Mcl-1, Bcl-2, Fas, Cyclin D1, survivin, and Myc [71,72,73,74,75]. 

## 3. TRIM8 and Stemness

Another aspect tightly linked to oncogenesis is the homeostasis of stem cells. Indeed, long-standing observations have noted that oncogenic pathways play a key role in stemness. In this context, it is not surprising that TRIM proteins also perform an important role in stem cell biology. TRIM28 functions as a transcriptional corepressor in orchestrating the primer binding site-mediated silencing of integrated Moloney murine leukemia virus (M-MLV) proviruses in mouse embryonic carcinoma and embryonic stem cells [76], while TRIM19 (PML) regulates the asymmetric division of hematopoietic stem cells via the PPAR-δ-FAQ pathway [77]. Again, TRIM3 regulates asymmetric cell division in normal neural progenitor cells as well as in Glioblastoma Stem-like Cells (GSC) [78] and maintains stem cell equilibrium by regulating active NOTCH1 nuclear transportation [79]. 

A recent study has shown that TRIM8 modulates the translocation of phosphorylated STAT3 into the nucleus through interaction with heat shock protein 90β (HSP90β), which interacts with STAT3, and consequently downregulates the transcription of Nanog in embryonic stem cells, suggesting that TRIM8 regulates the self-renewal or differentiation of stem cells [63]. 

TRIM8 also has a role in maintaining stemness and self-renewing capabilities of GSC, which contribute to the rapid growth, therapeutic resistance, and clinical recurrence of these aggressive tumours [80,81]. TRIM8 expression is highly correlated with GSC markers (CD133 and NESTIN), as well as the stem cell transcription factors SOX2 and c-MYC, sustaining cell proliferation by activating STAT3, through the suppression of its direct regulator PIAS3 [66]. In glioblastoma multiforme (GBM), STAT3 has been well documented as a facilitator of tumour cell proliferation, invasion, and angiogenesis and is an essential factor that maintains the stem cell phenotype of GSCs. On the contrary, the downregulation of TRIM8 correlates with the loss of stemness and the enhancement of GSC differentiation, with the reduction of phosphorylated STAT3 levels.

Although the mechanisms that activate TRIM8 or cause its increased expression in GSCs are not established, it has been previously reported that STAT3 could directly activate TRIM8 transcription through the STAT3-responsive elements identified in the TRIM8 promoter region or that those could be accomplished through c-MYC and OCT1 (Organic Cationic Transporter 1). This data supports the role of TRIM8 as a potential oncogene in GBM through its regulation of PIAS3-STAT3 and GSC stemness, rather than as a tumour suppressor, as might be expected by its frequent deletion.

The TRIM8/STAT3 positive feedback loop in GBM provides new insight into the regulation of STAT3 and offers new opportunities for the development of treatments that impair stemness in GSC and potentially in other cancers that show TRIM8 overexpression. That is very interesting since STAT3 activation sustains tumour growth properties, including the support of stemness, in diverse cancer types including those of the breast, prostate, and brain and targeting STAT3 or its crucial upstream activators could result in the disruption of GSC maintenance and this pathway could represent a potential therapeutic target [80,82,83,84,85,86,87]. Indeed, by targeting either TRIM8 or STAT3, researchers would be able to disrupt their positive feedback loop and attenuate the self-renewal capacity and tumour propagation of GBM.

## 4. TRIM8 and Its Emerging Role in Innate Immunity

Another important cellular function in which TRIM8 is involved is the regulation of innate immunity, an aspect again closely linked to cancer. 

Chartagena et al. (2009) reports a systematic analysis of *TRIM* gene expression in human primary lymphocytes and monocyte-derived macrophages in response to interferons (IFNs, type I and II) or following the FcγReceptor-mediated activation of macrophages during antiviral immune responses [88]. IFNs are the main mediators of innate immunity against viral infection, by upregulating the expression of many antiviral effectors within cells. The authors demonstrated that TRIM family members are critical immune regulatory proteins, and induction by IFNs has been reported for TRIM5, TRIM8, and TRIM19/PML proteins. 

In particular, the critical role in antiviral immunity of TRIM8 has been demonstrated to be conserved in evolution. Huang et al. (2016) report the phylogenetic analysis of TRIM8 from different vertebrate species (mammalian, birds, anphibia, fish) and the amino acid alignment shows that the RING and the B-box domains are highly conserved throughout evolution. The authors investigated the roles of the TRIM8 homolog from commercially important farmed fish species in China and Southeast Asian countries during virus infection. The outbreak of iridoviral and nodaviral diseases always caused extremely high mortality at different developmental stages and heavy economic losses in grouper aquaculture [89]. They report that TRIM8 over-expression inhibited fish iridovirus and noda virus replication, and was able to regulate the expression of pro-inflammatory cytokines and interferon-related signaling molecules. 

Since the *TRIM* expansion in evolution shows striking parallels with the development of innate and adaptive immune traits, it has been speculated that the TRIM family may have expanded to provide regulatory mechanisms for the increasingly complex immune system.

However, the manner in which different TRIMs regulate the immune response is complex. The same TRIMs may have different, multiple, or even opposite functions in different signaling pathways and some functions may be species-specific and cell type-specific.

Moreover, correct spatio-temporal regulation of immune signaling is critical to adequately respond to pathogen infection, maintaining immune balance, while preventing diseases characteristic of aberrant immune responses, such as chronic inflammatory diseases, autoimmune disorders, and cancer.

## 5. Concluding Remarks

TRIM8, in conclusion, represents a multifaced key factor that is able to perform different functions including cell growth, innate immunity, inflammation, and stem cell regulation. All these functions may seem counteractive, but several publications have shown that the regulation of innate immunity and inflammation are tightly linked to carcinogenesis and are now both beginning to be linked to stemness as well. Indeed, several publications have shown that classic tumour suppressors such as *p53* and *pRb* have emerging roles in the regulation of stemness [90,91]. In addition to this, genes generally known for their key roles in stem cell biology, for example, Nanog, appear to be deregulated in a number of cancers [92,93,94]. Therefore, the different functions in which TRIM8 is involved are probably dependent on the cellular context and/or on the different oncogenic stimuli. For example, it should be noted that many of the experiments demonstrating a role for TRIM8 in oncogenesis were conducted in a p53-null or p53-inactivated background, a caveat that ought to be taken into account in the understanding of a role for TRIM8 in cancer generation/progression. 

It might be supposed that TRIM8 signaling pathways exert tumour suppressor effects in normal cells (i.e., stem cells) and early carcinomas. As tumours develop and progress, these protective and cytostatic effects of TRIM8 are often lost. TRIM8 signaling may then switch to promote cancer progression, invasion, and tumour metastasis. 

While there is still much to be clarified in terms of the TRIM8 roles in the cell, the understanding of the complicated relationship between inflammation, immunity, stemness, and cancer may hold the key to more successful future therapies aimed at blocking the pro-oncogenic arm of the TRIM8 signalling pathway without affecting its tumour suppressive effects.
